# An audit of the initial resuscitation of severely ill patients presenting with septic incomplete miscarriages at a tertiary hospital in South Africa

**DOI:** 10.1186/s12884-015-0510-7

**Published:** 2015-04-02

**Authors:** Hennie Lombaard, Sumaiya Adam, Jennifer Makin, Patricia Sebola

**Affiliations:** Maternal and Fetal Medicine Unit, Department Obstetrics and Gynecology, Steve Biko Academic Hospital, University of Pretoria, Pretoria, South Africa; Department of Obstetrics and Gynecology, University of Pretoria, Pretoria, South Africa; Medical Research Council Maternal and Infant Health Strategies Research Unit, University Pretoria, Pretoria, South Africa; Department of Obstetrics and Gynecology, Steve Biko Academic Hospital, University of Pretoria, Pretoria, South Africa

**Keywords:** Septic incomplete miscarriage, Maternal near miss, Maternal death, Shock index, SAMM

## Abstract

**Background:**

Septic incomplete miscarriages remain a cause of maternal deaths in South Africa. There was an initial decline in mortality when a strict protocol based approach and the Choice of Termination of Pregnancy Act in South Africa were implemented in this country. However, a recent unpublished audit at the Pretoria Academic Complex (Kalafong and Steve Biko Academic Hospitals) suggested that maternal mortality due to this condition is increasing. The objective of this investigation is to do a retrospective audit with the purpose of identifying the reasons for the deteriorating mortality index attributed to septic incomplete miscarriage at Steve Biko Academic Hospital.

**Methods:**

A retrospective audit was performed on all patients who presented to Steve Biko Academic Hospital with a septic incomplete miscarriage from 1^st^ January 2008 to 31^st^ December 2010. Data regarding patient demographics, initial presentation, resuscitation and disease severity was collected from the “maternal near-miss”/SAMM database and the patient’s medical record. The shock index was calculated for each patient retrospectively.

**Results:**

There were 38 SAMM and 9 maternal deaths during the study period. In the SAMM group 86.8% and in the maternal death group 77.8% had 2 intravenous lines for resuscitation. There was no significant improvement in the mean blood pressure following resuscitation in the SAMM group (p 0.67), nor in the maternal death group (p 0.883). The shock index before resuscitation was similar in the two groups but improved significantly following resuscitation in the SAMM group (p 0.002). Only 31.6% in the SAMM group and 11.1% in the maternal death group had a complete clinical examination, including a speculum examination of the cervix on admission. No antibiotics were administered to 21.1% in the SAMM group and to 33.3% in the maternal death group.

**Conclusion:**

The strict protocol management for patients with septic incomplete miscarriage was not adhered to. Physicians should be trained to recognise and react to the seriously ill patient. The use of the shock index in the identification and management of the critically ill pregnant patient needs to be investigated.

## Background

A septic incomplete abortion (S-ICA) is an infection of the uterus or its contents that may spread haematologically to the pelvis, peritoneal cavity and vital organs. It manifests with pyrexia, associated with an offensive vaginal discharge, lower abdominal pain and signs of sepsis. Any abortion may produce septic sequelae, but the consequences of sepsis are usually much more severe following an unsafe termination of pregnancy.

According to the National Committee of the Confidential Enquiries into Maternal Deaths (NCCEMD) Saving Mothers report for 2005-2009 [1] abortion still remained one of the causes of maternal deaths in South Africa. Unsafe abortions accounted for 3.4% of maternal deaths [1]. Prior to the introduction of the Choice of Termination of Pregnancy Act No 92 of 1996, 34% of hospital admissions for septic abortions were due to unsafe abortions [2-4]. With the introduction of the Act there was a significant decline in the maternal deaths from unsafe abortion. Furthermore, a strict protocol for managing women with unsafe abortion led to a reduction in maternal mortality from unsafe abortions between 2002 and 2004 [5]. However, an unpublished audit conducted in the Pretoria Academic Complex (Kalafong and Steve Biko Academic Hospitals) showed a rise in the mortality index for the period 2008-2009 compared to 1997-1998.

Early recognition and management of sepsis has been shown to be effective in reducing morbidity and mortality. Patients are triaged according to their pulse and blood pressure. However, the use of the patients’ vital statistics alone has been shown to be unreliable in identifying patients with hypovolemic shock. The shock index (heart rate/systolic blood pressure) is a simple, valuable tool in identifying a shocked patient, even when the patient’s peripheral blood pressure may be within the normal range [6,7].

The aim of this study is to retrospectively audit the severe acute maternal morbidity (SAMM) and maternal deaths due to S-ICA and to identify the reasons for the deterioration in the mortality index attributed to it. Women who survive life threatening conditions (SAMM) arising from complications related to pregnancy have common aspects to those who die of such complications [8,9].

## Methods

A retrospective study was conducted at Steve Biko Academic Hospital in Pretoria, South Africa. We recruited all patients who presented with a septic incomplete abortion resulting in severe acute maternal morbidity (SAMM) or maternal near miss, or a maternal death during the period January 2008 to December 2010.

A septic incomplete abortion was defined as the loss of a fetus before 20 weeks gestation or a fetus weighing less than 500 g. Patients presented with either pyrexia (temperature >37.3°C) or an offensive vaginal discharge.

A severe acute maternal morbidity (SAMM) or maternal near miss is a “woman who nearly died, but survived a complication that occurred during pregnancy, childbirth or within 42 days of termination of pregnancy” [8]. The maternal near miss or SAMM was classified according to the WHO criteria [9].

Approval for this study was obtained from the Research Ethics Committee of the University of Pretoria, Ethics Approval number 5/2012. Permission was obtained from the CEO of Steve Biko Academic Hospital to access the patient files. Data regarding patient demographics, initial presentation, resuscitation and disease severity was collected retrospectively from the maternal near miss database which is available to the Department of Obstetrics and Gynaecology, and the patient’s medical record. The shock index (SI = pulse rate/ systolic blood pressure) was calculated for each patient retrospectively using the data from the medical records.

Continuous data was analysed using paired sample tests. Comparison of the first and second set of patients’ vital statistics and comparison between the severe acute maternal morbidity and the maternal death group was made using the Wilcoxon Rank test. The Mann-Whitney U test was used to compare the severe acute maternal morbidity and the maternal death group.

## Results

Thirty eight (9%) of 417 SAMM and nine (30%) of the 30 maternal deaths in the period January 2008 to December 2010 were attributed to septic incomplete abortions (S-ICA). The average time from initial intervention (e.g. misoprostol) or symptoms (vaginal bleeding) to presentation to hospital was 6.7 days for the SAMM group and 7.5 days for the maternal death group. The patients with SAMM spent an average of 10 days in hospital. Standard resuscitation of a patient with an S-ICA includes intravenous lines (two wide bore intravenous lines if the patient has hypovolaemic shock), a urinary catheter, oxygen via facemask and blood transfusion as required (Table 1).

**Table 1:** **Patient demographics and initial resuscitation**

		**SAMM (38)**	**Maternal death (9)**
**Age (years)**	**Mean**	29	32
	**Range**	21 - 43	22 - 41
**Parity**	**Median**	2	2
	**Range**	0 - 4	0 - 4
**HIV status**	**Negative**	11 (28.9%)	3 (33.3%)
	**Positive**	12 (31.6%)	3 (33.3%)
	**Unknown**	15 (39.5%)	3 (33.3%)
**Days from symptoms to presentation**	**Mean**	6.7	7.5
	**Range**	2 - 15	1 - 21
**Average duration of admission**		10	
**Resuscitation**	**2 ivi lines**	33 (86.8%)	7 (77.8%)
	**Urinary catheter**	22 (57.9%)	7 (77.8%)
	**Oxygen**	3 (7.9%)	6 (66.7%)
	**Blood products**	7 (22.6%)	9 (100%)
		(excluded 7 patients who had Hb > 8)	

There was no significant improvement in mean blood pressure following resuscitation in the SAMM group (p 0.67), nor in the maternal death group (p 0.883). Also, the patients in the SAMM group presented with a lower mean BP than patients in the maternal death group (p 0.244).

The shock index (SI = pulse rate/systolic blood pressure) on presentation was similar in the two groups (p 0.432), whereas the second SI differed significantly different in them (p <0.001). However, the shock index deteriorated in the maternal death group (p 0.95), whereas it improved in the SAMM group (p 0.002) (Table 2).

**Table 2:** **Initial resuscitation (1) versus patient condition on arrival in theatre or just prior to death (2)**

		**SAMM (38)**	**Maternal death (9)**
**Mean BP - 1 (mmHg)**	**Mean**	71.2	85.7
	**Range**	41 – 107	48 – 135
	**SD**	15.4	30.7
**Mean BP - 2 (mmHg)**	**Mean**	73	66.8
	**Range**	43 – 95	33 – 86
	**SD**	10.5	20.4
**SI – 1**	**Mean**	1.2	1.3
	**Range**	0.67 - 2.14	0.73 - 1.78
	**SD**	0.35	0.37
**SI – 2**	**Mean**	1.07	1.65
	**Range**	0.73 - 1.6	1.0 - 2.0
	**SD**	0.26	0.48
**SI ≥1 – 1**		27 (71.1%)	7 (77.8%)
**SI ≥1 - 2**		23 (60.5%)	9 (100%)

Mean BP – 1 - at presentation.

Mean BP – 2 - On arrival in theatre or last vitals before death.

SI – 1 - at presentation.

SI – 2 - On arrival in theatre or last vitals before death.

Evaluation of patient examination showed that the most neglected system was the genital tract which excluded a speculum examination of the cervix. Only 12 (31.6%) patients in the SAMM group and one (11.1%) patient in the maternal death group were fully examined on presentation.

The systemic examination showed that the circulatory system was most commonly affected with 21 (55.3%) patients in the SAMM group presenting with haemorrhagic shock and 2 (5.3%) patients presenting with septic shock. In the maternal death group 7 (77.8%) patients presented with haemorrhagic shock and 2 (22.2%) with septic shock. One (2.6%) patient in the SAMM and one (11.1%) in the maternal death group presented with respiratory failure. Four (10.5%) patients in the SAMM group and one (11.1%) in the maternal death group had renal failure. Sixteen (42.1%) patients in the SAMM group and 2 patients (22.2%) in the maternal death group presented with DIC (disseminated intravascular coagulation).

Eight patients (21.1%) with SAMM and 3 (33.3%) of the maternal deaths received no antibiotics during the initial resuscitation. The rest of the patients received either a single dose of cefazolin or a combination of cefazolin and metronidazole, or cefazolin, metronidazole and gentamycin.

Twenty (60.5%) patients with SAMM and 2 (22.2%) of the maternal deaths had an evacuation of the uterus. Fifteen patients (39.5%) in the SAMM group and 2 (22.2%) in the maternal death group had total abdominal hysterectomies. Five (55.5%) patients demised prior to any surgical intervention.

The shock index was evaluated retrospectively to judge its value in predicting the need for blood transfusion and for prognosticating severe sepsis and death (Table 3).

**Table 3:** **The Predictive value of the shock index**

**Shock index – 1 to predict the need for blood transfusion**			
**SI-1 > 0.9**	**Transfusion**	29	Sensitivity 82.9%
	**No transfusion**	9	
**SI-1 ≤ 0.9**	**Transfusion**	6	Specificity 25%
	**No transfusion**	3	
**Shock Index – 2 to predict mortality**			
**SI-2 ≥ 1**	**TAH**	11	Sensitivity 100%
	**SAMM**	23	
	**Maternal death**	9	
	**Organ dysfunction**	32 (19 multi-organ)	
**SI-2 < 1**	**TAH**	5	Specificity 39.5%
	**SAMM**	15	
	**Maternal death**	0	
	**Organ dysfunction**	15 (4 multi-organ)	

SI-1 – shock index on admission.

SI-2 – shock index on arrival in theatre or last vitals before death.

Only 11 (28.9%) patients were discharged with a form of contraception.

## Discussion

Abortion remains a cause of maternal death in the developing world, with sepsis being a major contributor [10,11]. Morbidity and mortality due to septic abortions are preventable [12]. Despite the introduction of the Choice of Termination of Pregnancy Act No. 92 of 1996 [2], and of the strict protocol management approach [5], we still have a high maternal morbidity and mortality due to septic incomplete abortions. The mortality index due to septic incomplete abortions in our study was 24%, which is four-fold that reported in the Saving Mothers Report of 2008-2010 [1].

The patients in our study cohort were young but never the less suffered from the complications of septic incomplete abortions. Complications were aggravated by delay in presenting to hospital. With a mean delay of 6.7 – 7.5 days. Although we were unable to obtain adequate information regarding the reasons for the delay, we have to consider the possibility of unwanted pregnancies and unsafe terminations of pregnancies.

Patients with septic incomplete abortions present with sequelae of sepsis as well as varying degrees of haemorrhage. Early and adequate fluid and antibiotic resuscitation improves outcomes in patients with septic incomplete abortions [13,14]. In the 2008-2010 Saving Mothers Report [1] delay in initiating critical care due to an overburdened service, failure to make a diagnosis and to recognise the severity of the condition, and substandard resuscitation were identified as the most frequent causative factors in maternal deaths due to septic incomplete abortions.

Most patients in this study presented in shock with the mean shock index on presentation ranging from 1.2 to 1.3. However there was a significant improvement in the second shock index in the SAMM group. This improvement was not seen in the patients who died. Lack of improvement may either be due to sub-optimal resuscitation or disease so severe that it became resistant to resuscitation efforts.

The recommended antibiotic regime in the unit for the management of septic incomplete abortions is cefuroxime, metronidazole and gentamycin. This was not followed and may have contributed to the high morbidity and mortality. The lack of early and adequate antibiotic administration may also be due to the lack of recognition of disease severity.

Organ failure in critically ill patients is associated with a higher mortality rate [15]. In our cohort all the patients had at least one organ system affected, and 23 (48.9%) patients had multi-organ failure. The organ systems most commonly affected were the circulatory, respiratory, renal and haematological systems.

Early recognition and treatment of sepsis and its effect on short-term survival has long been recognised. Reliable indicators may assist in effective triaging and goal-directed management of patients. The use of the patients’ heart rate and blood pressure alone has been shown to be unreliable in identifying patients with hypovolaemic shock. The shock index has thus been suggested as a marker to predict the severity of hypovolaemic shock. It is also used in evaluating patients with sepsis. The shock index is a valuable tool that can raise suspicion even when the pulse and blood pressure are within normal range [6,7].

Furthermore, the shock index better identifies patients with increased transfusion requirements and early mortality. A linear relationship has been described between haemorrhage and an increasing shock index. Rady et al showed that a shock index >0.9 was associated with a need for immediate admission and transfusion [7]. The shock index has a high sensitivity and specificity when used as a predictor of death in patients with severe sepsis or septic shock [16,17]. Shah Jahan et al suggested that cut-off of ≥1.0 for the second shock index following resuscitation predicted mortality with a sensitivity of 80.8% and a specificity of 79.2% [18]. This study was too small to evaluate the use of the shock index in prognosticating the need for transfusion or mortality. The data was incomplete for some patients as it was a retrospective study. Also we were unable to obtain enough information regarding the delay in presentation to hospital, other medical care sought or unsafe interventions. The shock index was applied retrospectively so we remain uncertain of its clinical utility in the critically ill patient with a septic abortion.

## Conclusions

The strict protocol management of septic incomplete miscarriages was not adhered to. Physicians should be trained to recognise and react to the seriously ill patient. A protocol of aggressive fluid and antibiotic therapy should be reinforced. The use of the shock index in the identification and management of the critically ill pregnant patient needs to be investigated.
